# Oxidative Stress Mediates Vascular Tortuosity

**DOI:** 10.3390/antiox10060926

**Published:** 2021-06-07

**Authors:** Toshio Fumoto, Shouhei Kinoshita, Takao Sasaki, Norihito Shimamura, Hiroki Ohkuma

**Affiliations:** Department of Neurosurgery, Graduate School of Medicine, Hirosaki University, 5-Zaifuchou, Hirosaki, Aomori 036-8562, Japan; kinoshita@hirosaki-u.ac.jp (S.K.); sasakisasaki@hirosaki-u.ac.jp (T.S.); shimab@hirosaki-u.ac.jp (N.S.); ohkuma@hirosaki-u.ac.jp (H.O.)

**Keywords:** oxidative stress, vascular tortuosity, apple polyphenol

## Abstract

Vascular tortuosity is associated with various disorders and is being increasingly detected through advances in imaging techniques. The underlying mechanisms for vascular tortuosity, however, remain unclear. Here, we tested the hypothesis that oxidative stress mediates the generation of tortuous vessels. We used the bilateral common carotid artery (CCA) ligation model to induce vascular tortuosity. Both young and adult rats showed basilar artery tortuous morphological changes one month after bilateral CCA ligation. These tortuous changes were permanent but more pronounced in the adult rats. Microarray and real-time PCR analysis revealed that these tortuous changes were accompanied by the induction of oxidative stress-related genes. Moreover, the indicated model in rabbits showed that tortuous morphological changes to the basilar artery were suppressed by antioxidant treatment. These results are highly suggestive of the significance of oxidative stress in the development of vascular tortuosity. Although further studies will be needed to elucidate the possible mechanisms by which oxidative stress enhances vascular tortuosity, our study also points toward possible prophylaxis and treatment for vascular tortuosity.

## 1. Introduction

Vascular abnormality predisposes to a wide variety of diseases. Vascular tortuosity is observed in virtually every tissue and in almost all types of blood vessels, from large arteries and veins to capillaries. Tortuous vessels are sometimes asymptomatic but are increasingly being detected through advances in imaging techniques. Vascular tortuosity has been shown to be associated with advancing age, female sex, and cardiovascular risk factors such as diabetes and hypertension. However, the underlying mechanisms for the initiation and progression of tortuous vessels are poorly understood [1,2]. Blood pressure and blood flow, important contributors to hemodynamic stress, have been shown to be associated with vascular tortuosity [1]. In addition, the control of hemodynamic stress itself induces vascular tortuosity in animal models [3,4]. Therefore, hemodynamic stress is thought to be a critical factor for the induction of vascular tortuosity. Reactive oxygen species mediate a wide variety of vascular functions in both physiological and pathological conditions. It is well established that hemodynamic stress induces reactive oxygen species in vascular walls [5,6]. However, the role of oxidative stress in vascular tortuosity is poorly understood.

Vascular tortuosity has also been shown to be associated with some genetic syndromes. Loeys–Dietz syndrome (LDS) and arterial tortuosity syndrome (ATS) are genetic disorders associated with arterial tortuosity that include tortuous cerebral arteries [7,8,9]. LDS is an autosomal dominant genetic disorder due to activating mutations in *TGFBR1*, *TGFBR2*, *SMAD3*, *TGFB2,* or *TGFB3* [10,11,12,13]. ATS is an autosomal recessive genetic disorder due to a mutation in *SLC2A10* encoding for the glucose transporter type 10 (GLUT10) [9]. GLUT10 has been shown to facilitate dehydroascorbic acid transport and protect against oxidative stress [14]. In addition, the *SLC2A10* mutation has been shown to lead to increased cellular oxidative stress and elevated transforming growth factor β (TGF-β) signaling in fibroblasts from ATS patients [15]. Thus, oxidative stress is presumed to be an upstream mediator of TGF-β signaling in the context of vascular tortuosity. Here, we examined the role of oxidative stress in the generation of tortuous vessels.

## 2. Materials and Methods

### 2.1. Animals

Male Sprague Dawley rats (one-year-old, 750–950 g, and three-month-old, 430–500 g) from CLEA Japan, Inc. (Tokyo, Japan) and female Japanese white rabbits (six-month-old, 3.8–4.5 kg) from Kitayama Labes Co., Ltd. (Ina, Japan) were used in this study. The animals were maintained under standard laboratory conditions (25 ± 2 °C, 12 h light/dark cycle) and allowed free access to water and standard pellet chow.

### 2.2. Animal Surgery

The animals were anesthetized using a combination anesthetic (Rat: medetomidine; 0.3 mg/kg, midazolam; 4.0 mg/kg, butorphanol; 5.0 mg/kg, Rabbit: medetomidine; 0.5 mg/kg, midazolam; 2.0 mg/kg, butorphanol; 0.5 mg/kg). Both CCAs were exposed via a midline cervical incision and ligated with a suture under deep anesthesia. For the sham operation, the CCAs were exposed without ligation.

### 2.3. Experimental Design

The animals were randomly divided into each group. In the rabbit experiment, the apple polyphenol (AP) group was given apple polyphenol orally (Apple Phenon: Asahi Breweries, Ltd., Tokyo, Japan, 125 mg/kg/day, dissolved in 2 mL water) beginning at two days before ligation and subjected to bilateral CCA ligation. The Ligation group was given the same volume of water and subjected to bilateral CCA ligation. The Sham group was subjected to the same procedure as the Ligation group but without ligation.

### 2.4. India Ink Angiography

Four weeks after the ligation, the rats were transcardially perfused with PBS, 4% paraformaldehyde, and 3.5% gelatin India ink solution under deep anesthesia. After 24 h of refrigeration at 4 °C, the brains were removed, and high-resolution pictures were taken.

### 2.5. Microarray Analysis

Four weeks after the ligation, the rats were transcardially perfused with PBS under deep anesthesia. The brains were removed, and high-resolution pictures were taken. The basilar arteries were collected and stored at −80 °C. Total RNA was extracted using TRIzol reagent (Thermo Fisher Scientific Inc., Waltham, MA, USA) and a Pure Link RNA Micro Kit (Thermo Fisher Scientific Inc., Waltham, MA, USA). The quality and quantity of RNA were evaluated using an Agilent 2100 Bioanalyzer (Agilent Technologies, Inc., Santa Clara, CA, USA) and a Nanodrop spectrophotometer (Thermo Fisher Scientific Inc., Waltham, MA, USA). Equal amounts of RNA from four rats in each group were pooled. The cDNA was synthesized from 50 ng RNA and labeled using a GeneChip WT PLUS Reagent Kit (Thermo Fisher Scientific Inc., Waltham, MA, USA). Samples were hybridized to a Clariom S Assay, rat (Thermo Fisher Scientific Inc., Waltham, MA, USA). The arrays were scanned with a GeneChip Scanner 300 7G (Thermo Fisher Scientific Inc., Waltham, MA, USA) and analyzed using the Microarray Data Analysis Tool Ver. 3.2 (Filgen, Inc., Nagoya, Japan).

### 2.6. Reverse Transcription and Quantitative PCR

Total RNA was isolated as described above. The cDNA was synthesized using an iScript gDNA Clear cDNA Synthesis Kit (Bio-Rad Laboratories, Inc., Hercules, CA, USA) and amplified by real-time PCR using the iTaq Universal SYBR Green Supermix (Bio-Rad Laboratories, Inc., Hercules, CA, USA) and the CFX Connect real-time PCR system (Bio-Rad Laboratories, Inc., Hercules, CA, USA). The sequences of the PCR primers were described in Table 1.

### 2.7. Elastica van Gieson (EVG) Staining and Immunostaining

At 12 weeks after ligation, rabbits were transcardially perfused with PBS and 4% paraformaldehyde under deep anesthesia. The brains were removed, and high-resolution pictures were taken. After overnight fixation using the same fixative, the samples were embedded in paraffin. The specimen was cut into 6 μm thick coronal sections. After deparaffinization and rehydration, sections were stained using standard histological dyes for EVG staining (MUTO PURE CHEMICALS CO., LTD., Tokyo, Japan). Antigen retrieval was performed using proteinase K. Immunohistological staining was performed, as previously described [16]. Briefly, sections were treated with BLOXALL (Vector Laboratories Inc., Burlingame, CA, USA) to quench endogenous peroxidase activity, and blocked using normal serum and an Avidin/Biotin Blocking Kit (Vector Laboratories Inc., Burlingame, CA, USA). The sections were then incubated with antibodies, Vectastatin Elite ABC Kit (Vector Laboratories Inc., Burlingame, CA, USA), and ImmPACT DAB (Vector Laboratories Inc., Burlingame, CA, USA). The following antibodies were used: mouse anti-nitrotyrosine monoclonal antibody (Merck Millipore, Burlington, MA, USA), mouse anti-interleukin 1 beta monoclonal antibody (Cloud-Clone Corp., Katy, TX, USA), goat anti-aquaporin 1 polyclonal antibody (Santa Cruz Biotechnology, Dallas, TX, USA), rabbit antioxidized low-density lipoprotein receptor 1 polyclonal antibody (abcam, Cambridge, UK). After counterstaining with hematoxylin, slides were observed using a light microscope (Olympus, Tokyo, Japan). For immunofluorescence staining, sections were blocked using BlckAid blocking solution (Thermo Fisher Scientific Inc., Waltham, MA, USA) and incubated with mouse anti-fibulin 5 monoclonal antibody (abcam, Cambridge, UK) or mouse anti-8 Hydroxyguanosine antibody (abcam, Cambridge, UK), and subsequently with Alexa Fluor 594 conjugated goat anti-mouse IgG antibody (Thermo Fisher Scientific Inc., Waltham, MA, USA).

After nuclear staining with DAPI, slides were observed using a fluorescent microscope and analyzed by BZ-X analyzer (KEYENCE, Osaka, Japan).

### 2.8. Measurement of Tortuosity

The tortuosity of the basilar artery was determined using high-resolution pictures taken before RNA extraction for rat or immunohistological staining for rabbit, and the previously described index [4]. Briefly, the tortuosity index (TI) is defined as TI = (Lc−Ls)/Ls, in which Lc is the curve length on the centerline between the two ends of the BA, and Ls is the straight-line distance between the two ends of the BA.

### 2.9. Statistical Analysis

All quantitative analyses were performed in a blinded fashion. The tortuosity index of rat and quantitative PCR were analyzed by the Mann–Whitney U test. using StatPlus software. The tortuosity index of rabbit was initially analyzed by the Shapiro–Wilk test and then analyzed by Kruskal–Wallis tests, followed by the Steel–Dwass test for multiple comparisons using JMP13 software. The data were presented as means and SD. Statistical significance was defined as *p* < 0.05.

## 3. Results

### 3.1. Bilateral CCA Ligation Induces Vascular Tortuosity in Rats

**Figure 1:**
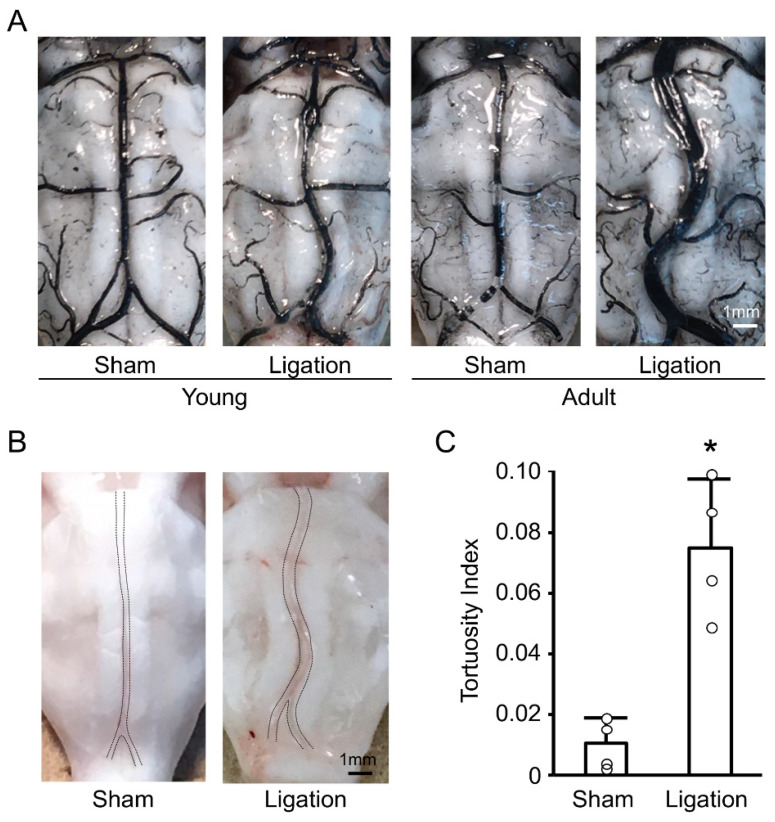
Bilateral common carotid artery (CCA) ligation induces basilar artery tortuosity in rats: (**A**) Representative India ink angiograms of young and adult rats at 4 weeks after bilateral CCA ligation; (**B**) Representative images of nonfixed basilar arteries of adult rat at 4 weeks after bilateral CCA ligation; (**C**) Tortuosity index of nonfixed basilar arteries of adult rats at 4 weeks after bilateral CCA ligation. *n* = 4 for each group. * *p* < 0.05.

We first examined the basilar artery morphology one month after bilateral CCA ligation, both in young and adult rats. As shown in Figure 1A, ligation induced basilar artery tortuosity both in young and adult rats, and these changes were more pronounced in adult rats. Next, we investigated whether these changes in adult rats were transient by using nonfixed animals. As shown in Figure 1B, basilar artery tortuosity was also observed in nonfixed animals. Quantitative analyses confirmed that bilateral CCA ligation induced permanent morphological changes to the basilar artery in adult rats within one month (Figure 1C).

### 3.2. Vascular Tortuosity Is Accompanied by the Induction of Oxidative Stress-Related Genes in Rats

**Figure 2:**
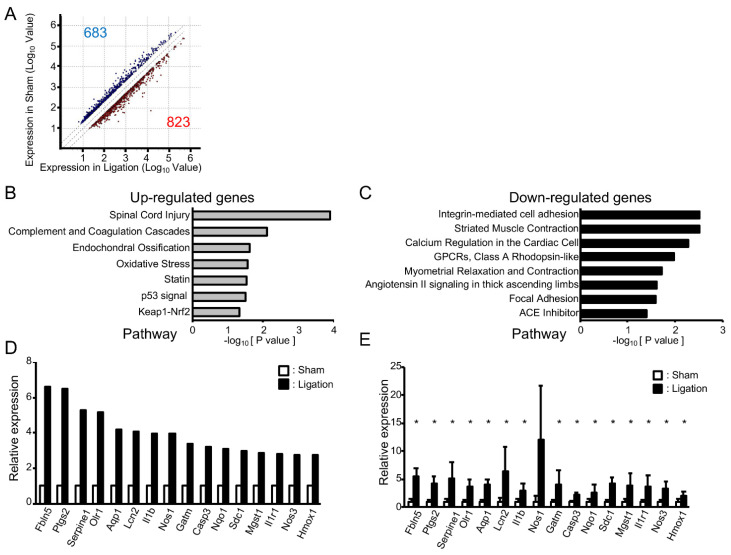
Vascular tortuosity is accompanied by upregulation of oxidative stress-related genes in rats: (**A**) Scattered plot of up- and downregulated genes in bilateral CCA ligated rat; (**B**,**C**) Pathways enriched among up- and downregulated genes in ligated rat; (**D**) Microarray analysis of oxidative stress-related genes that are upregulated in bilateral CCA ligated rat; (**E**) Real-time PCR analysis of oxidative stress-related genes. *n* = 4 for each group. Data are expressed as mean and SD. * *p* < 0.05.

To address the impact of oxidative stress on these morphological changes in adult rats, we next compared gene expression in the basilar artery of ligated rats with sham-operated rats using microarray analysis. A total of 23,188 genes were robustly expressed. As shown in Figure 2A, the expression of 823 genes was increased (>2.0-fold) and 683 genes decreased (<0.5-fold). Pathway analysis showed that seven pathways were significantly upregulated and eight pathways downregulated (Figure 2B,C). Consistent with the fact that the “oxidative stress” pathway was upregulated, a gene ontology (GO) analysis of upregulated genes showed GO terms “Response to oxidative stress”, “Cellular response to oxidative stress” and “Cellular response to superoxide” were also significantly upregulated (*p* = 2.69 × 10^−4^, 3.31 × 10^−3^, 1.70 × 10^−2^, respectively). For the GO term “Response to oxidative stress”, the expressions of 34 genes were increased at least 2.0-fold, and these genes included upregulated genes in “Oxidative stress”, “Cellular response to oxidative stress” and “Cellular response to superoxide”. Among them, the expression of 16 genes was upregulated at least 2.5-fold (Figure 2D). To validate the results of microarray analysis, we next examined the expression of these 16 genes by real-time PCR analysis. As shown in Figure 2E, expressions of all of these genes were higher in ligated animals and, among them, the expression of 15 genes was significantly upregulated in ligated rats.

### 3.3. Antioxidant Treatment Suppresses Vascular Tortuosity in Rabbits

**Figure 3:**
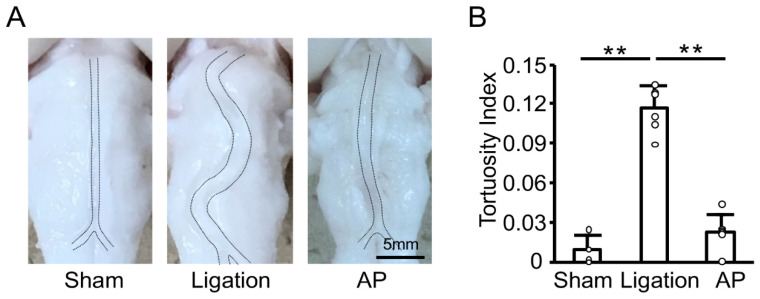
Antioxidant treatment suppresses vascular tortuosity in rabbits: (**A**) Representative images of fixed basilar arteries at 12 weeks after bilateral CCA ligation. AP: apple polyphenol treatment group. (**B**) Tortuosity index of fixed basilar arteries at 12 weeks after bilateral CCA ligation. *n* = 4 for Sham group. *n* = 6 for Ligation and AP group. ** *p* < 0.01.

To investigate the effect of oxidative stress further, we next examined the effect of antioxidant treatment on the basilar artery morphological change induced by bilateral CCA ligation in rabbits. We had previously established the oral administration of apple polyphenol in rabbits [17]. As shown in Figure 3A, bilateral CCA ligation induced basilar artery tortuosity, and apple polyphenol treatment suppressed this change. Quantitative analyses confirmed basilar artery tortuosity was significantly inhibited by antioxidant treatment (Figure 3B).

### 3.4. Antioxidant Treatment Suppresses the Induction of Oxidative Stress-Related Genes in Rabbits

**Figure 4:**
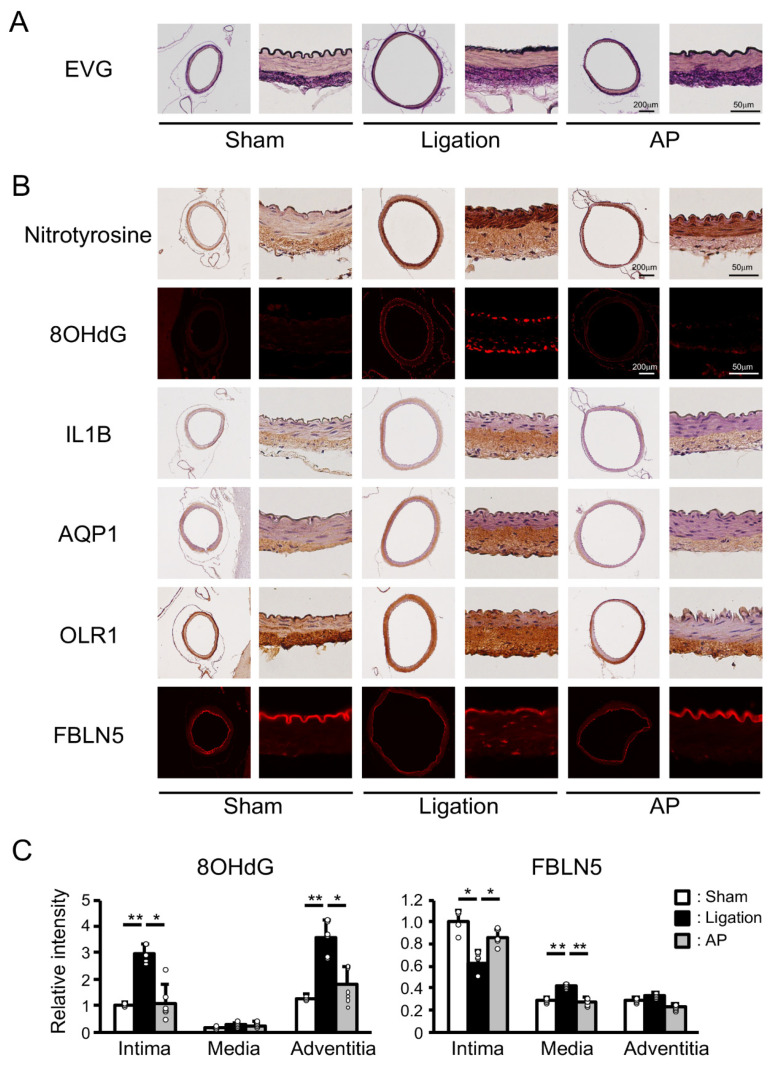
Effect of antioxidant treatment on vascular damage and oxidative stress-related genes in rabbits: (**A**) Representative images of EVG (Elastica van Gieson) staining. AP: apple polyphenol treatment group. (**B**) Representative images of immunostaining of oxidative stress-related genes. 8OHdG: 8-hydroxydeoxyguanosine; IL1B: interleukin 1 β; AQP1: aquaporin 1; OLR1: oxidized low-density lipoprotein receptor 1; FBLN5: fiburin 5. (**C**) Relative intensity of immunofluorescence staining. n = 4 for Sham group. n = 6 for Ligation and AP group. ** *p* < 0.01, * *p* < 0.05.

To gain some mechanistic insight into these protective effects of apple polyphenol, we performed Elastica van Gieson (EVG) staining of the basilar artery. Well-ordered internal elastic lamina was seen in sham-operated animals, while discontinuous elastic lamina was observed in ligated animals. Apple polyphenol treatment suppressed these changes (Figure 4A). We next performed immunostaining of the basilar artery. Nitrotyrosine and 8-hydroxydeoxyguanosine (8OHdG) are specific markers of oxidative stress in protein and DNA, respectively. Only slight nitrotyrosine staining was detected in the adventitia of sham-operated rabbits. In addition to the adventitia, clear staining was observed in the media of ligated animals. Apple polyphenol treatment suppressed this staining in both adventitia and media. Moreover, 8OHdG staining was induced in intima and adventitia by ligation, and these inductions were suppressed by apple polyphenol treatment (Figure 4B and Figure S1). Interleukin 1 β (IL1B), oxidized low-density lipoprotein receptor 1 (OLR1), aquaporin 1 (AQP1), and fibulin 5 (FBLN5) are bilateral CCA ligation-induced and oxidative stress-related genes, as seen in the rat experiment in this study. As shown in Figure 4B, expressions of IL1B were induced in adventitia by ligation, and these inductions were suppressed by apple polyphenol treatment. AQP1 showed similar trends. In contrast, expressions of OLR1 were spread from adventitia to media by the ligation, and these inductions were suppressed by apple polyphenol treatment. While FBLN5 was distributed only in the intima of sham-operated animals, FBLN5 staining diminished in the intima and was induced in the media and adventitia after ligation, and apple polyphenol treatment suppressed these changes. Quantitative analyses of immunofluorescence staining confirmed these effects of apple polyphenol (Figure 4C and Figure S2).

## 4. Discussion

In this study, we examined the role of oxidative stress in the development of vascular tortuosity using two models.

Using the rat bilateral CCA ligation model, we demonstrated rapid and persistent tortuous morphological changes to the basilar artery here. A previous study showed that the rat basilar artery exhibited tortuous morphological changes at six months after bilateral CCA ligation, and these changes were more pronounced in adult animals [3]. However, the short-term effects of bilateral CCA ligation on basilar artery morphology are not clear. In this study, we demonstrated basilar artery tortuous morphological changes, one month after bilateral CCA ligation. These changes were permanent and more pronounced in adult rats, similar to the previously reported long-term experiment [3]. Although rat bilateral CCA ligation is widely used as a model for hypoperfusion-related neurodegenerative diseases [18], this model has also proven useful for the study of vascular tortuosity.

Using this model, we have presented these tortuous changes here as accompanied by the induction of oxidative stress-related genes. Pathway analysis showed that the “Oxidative stress” pathway was significantly upregulated and GO analysis showed that GO terms “Response to oxidative stress”, “Cellular response to oxidative stress” and “Cellular response to superoxide” were also significantly upregulated. These data suggest that oxidative stress is important for the development of vascular tortuosity.

In addition, this research has demonstrated that tortuous morphological changes to the basilar artery were suppressed by antioxidant treatment in the rabbit bilateral CCA ligation model. Previous studies have shown that bilateral CCA ligation induced a rapid increase and a continuous elevation of blood flow in the basilar artery, gradually increasing the basilar artery diameter as well as the progressive tortuosity of the basilar artery. These changes were followed by substantial internal elastic lamina fragmentation of the basilar artery at 12 weeks after ligation [3]. In this study, we confirmed basilar artery tortuous morphological changes at three months after bilateral CCA ligation and showed suppression of these changes by antioxidant treatment. These results further support the hypothesis that oxidative stress is important for the development of vascular tortuosity.

This study provides some clues for molecular mechanisms by which oxidative stress enhances vascular tortuosity. We have demonstrated here that *Serpine1* mRNA was robustly and significantly induced by bilateral CCA ligation in the rat. *Serpine1* was shown to be a direct target of the TGF-β signaling pathway [19]. In addition, we found a slight increase in the expression of CTGF (connective tissue growth factor), a widely accepted target of TGF-β signaling [20] in the ligated rat (1.5-fold). These data raise the possibility that increased oxidative stress could enhance vascular tortuosity via activation of the TGF-β signaling pathway. Further studies will be needed to elucidate the relationship between oxidative stress and TGF-β signaling in vascular tortuosity.

Among oxidative stress-related genes, *Fbln5* mRNA encoding fibulin 5 was most potently induced by bilateral CCA ligation in the rat. Fibulin 5 protein was decreased in the intima and increased in media and adventitia after ligation in the rabbit. These changes reflect degradation of the internal elastic lamina and de novo synthesis, respectively. Additionally, both changes were suppressed by antioxidant treatment. Fibulin 5 was initially identified by two groups as the protein involved in the phenotypic transformation of vascular smooth muscle cells and cardiovascular development, respectively [21,22]. In these reports, fibulin 5 was shown to be highly expressed in developing arteries and dramatically downregulated in adult arteries. However, intense focal expression was found at intercostal branching points in the thoracic aorta and induced by vascular injury, both conditions reflecting imposed hemodynamic stresses. In accordance with these reports, we have demonstrated here that fibulin 5 was induced under flow-induced hemodynamic stresses. Fibulin 5 was shown to play multiple biological roles under different circumstances. It was reported that fibulin 5 bound to extracellular superoxide dismutase and regulated the levels of the superoxide anion [23]. Fibulin 5 could play roles in defense against oxidative stress in bilateral CCA ligation models, as many genes with a defensive function against oxidative stress were induced. It was also shown that fibulin 5 inhibited vascular smooth muscle cell proliferation and migration [24]. Fibulin 5 may regulate vascular smooth muscle cells and thereby play some roles in bilateral CCA ligation models. It was also demonstrated that fibulin 5 played a pivotal role in elastogenesis. Fibulin 5 deficient mice showed various defects in elastic fiber, including loose skin, vascular abnormalities, emphysematous lung, and genital prolapse [25,26]. Fibulin 5 knockout mice had tortuous aortas with disorganized elastic lamellae, similar to mice deficient in genes involved in elastogenesis. By contrast, we showed here that fibulin 5 was induced at the time of elastic lamina degradation. Elastic fiber resynthesis may be involved in flow-induced vascular remodeling, whereby fibulin 5 might participate.

There are several limitations in the present study. First, we used two-dimensional images to determine vascular tortuosity in this study. Three-dimensional geometry may be better for detailed analysis. In addition, we could not clarify whether these effects of apple polyphenol are the direct consequence of its antioxidative property or not. Some oxidative stress-related genes induced in the present study are related to inflammation. Further detailed studies are needed to clarify the sources of oxidative stress, the cell types involved in the development of tortuosity, and the underlying molecular mechanisms. Moreover, increased blood pressure in the rat bilateral CCA ligation model is reported and hypertension is closely related to vascular remodeling. Further studies of blood circulation in this model per ser and in comparison with the other hypertensive and neurodegenerative models [27] are needed in the future.

## 5. Conclusions

In summary, our report shows that tortuous changes to the basilar artery were accompanied by the induction of oxidative stress-related genes and suppressed by antioxidant treatment. These results are highly suggestive of the significance of oxidative stress in the development of vascular tortuosity. Although further studies will be needed to clarify the mechanisms by which oxidative stress enhances vascular tortuosity, our study also points toward possible prophylaxis and treatment for vascular tortuosity.

## Figures and Tables

**Table 1:** Primer sequences for qPCR.

	Forward	Reverse	Gene Accession Number
*Actb*	5′-CATGAAGATCAAGATCATTGCTCCT-3′	5′-CTGCTTGCTGATCCACATCTG-3′	NM_031144.3
*Fbln5*	5′-CATGCGGCAAACAGGGCCTA-3′	5′-TATCCGCAGTCGGATCACGG-3′	NM_019153.3
*Ptgs2*	5′-TTCGCATTCTTTGCCCAGCA-3′	5′-TTAAGTCCACTCCATGGCCCAG-3′	NM_017232.3
*Serpine1*	5′-ATCGAGGTGAACGAGAGCGG-3′	5′-ATTGTCTCTGTTGGATTGTGCCG-3′	NM_012620.1
*Olr1*	5′-TTCTTCCACATGGTGGTGCC-3′	5′-TACCTGGAGTAACTGTGTCTGCTG-3′	NM_133306.2
*Aqp1*	5′-TCTGGCTACCACTGACCGGA-3′	5′-GTAGTCAATGGCCAGCAGGTG-3′	NM_012778.1
*Lcn2*	5′-TTCTCTGTTCCCACCGACCA-3′	5′-ACAGGAAAGATGGAGCGGCA-3′	NM_130741.1
*IL1b*	5′-AATAGCAGCTTTCGACAGTGAGG-3′	5′-TGGACAGCCCAAGTCAAGGG-3′	NM_031512.2
*Nos1*	5′-CGCTACGCGGGCTACAAGCA-3′	5′-GCACGTCGAAGCGGCCTCTT -3′	NM_052799.1
*Gatm*	5′-CAAGCCCACAATGGCTGACG-3′	5′-GCTCGAACTCGGTTGTCACG-3′	NM_031031.2
*Casp3*	5′-TGGAACGAACGGACCTGTGG-3′	5′-TACCTCGGCAGGCCTGAATG-3′	NM_012922.2
*Nqo1*	5′-GCGGTGAGAAGAGCCCTGAT-3′	5′-GGTCAGATTCGACCACCTCCC-3′	NM_017000.3
*Sdc1*	5′-GCCTGACCTTCGGAATCAGT-3′	5′-ATGACACCTCCCAGCACTTC-3′	NM_013026.2
*Mgst1*	5′-CCAACCCGGAAGACTGTGCT-3′	5′-AAGGTCGTTCAGGTGGGCTC-3′	NM_134349.3
*IL1r1*	5′-TGGCTGAAGAGCACAGAGGG-3′	5′-TCGTCTCATTCCGTGGGCTC-3′	NM_013123.3
*Nos3*	5′-ACTGGTATCCTCTTGGCGGC-3′	5′-GCACAGAAGTGCGGGTATGC-3′	NM_021838.2
*Hmox1*	5′-CCTGCTAGCCTGGTTCAAGATAC-3′	5′-TGAGTGTGAGGACCCATCGC-3′	NM_012580.2

## Data Availability

The microarray data were deposited in the Gene Expression Omnibus (GEO), with GEO accession number GSE174455.

## Supplementary Materials

The following are available on line at https://www.mdpi.com/article/10.3390/antiox10060926/s1, Figure S1: Effect of apple polyphenol on oxidative stress markers. Figure S2: Effect of apple polyphenol treatment on nitrotyrosine levels.
