# Supplementary material for: Oxidative Stress Mediates Vascular Tortuosity
Source: Antioxidants (Basel). 2021 Jun 7;10(6):926. doi: 10.3390/antiox10060926 (PMC8228074; doi:10.3390/antiox10060926)
Supplement: Supplementary file 1 [file antioxidants-10-00926-s001.zip › antioxidants-1204175-supplementary.pdf]

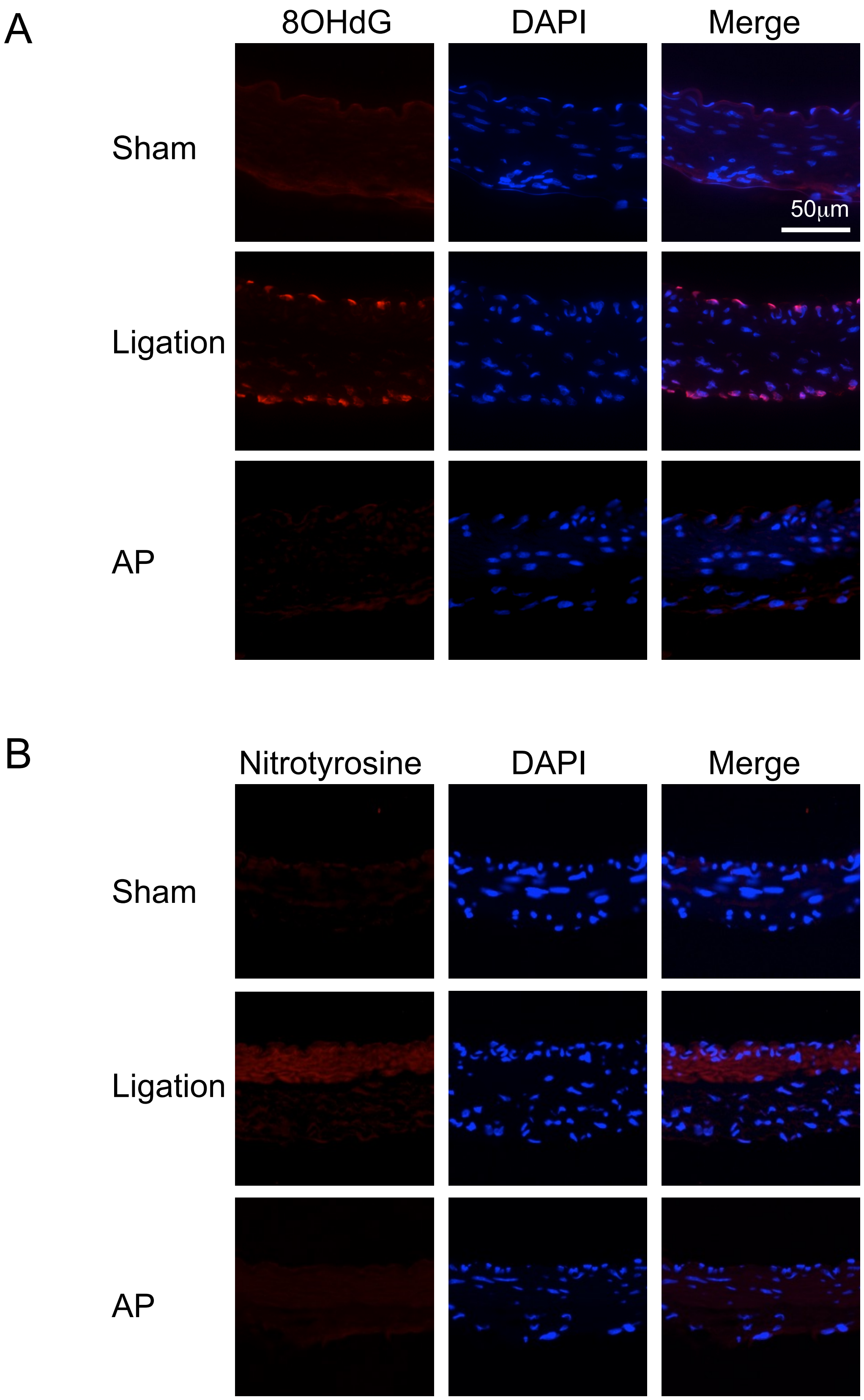

Supplemental Figure 1. Effect of apple polyphenol (AP) on oxidative stress markers.
(A) Representative images of immunofluorescence staining of 8-Hydroxydeoxyguanosine (8OHdG).
(B) Representative images of immunofluorescence staining of nitrotyrosine.
DAPI was used for nuclear staining.

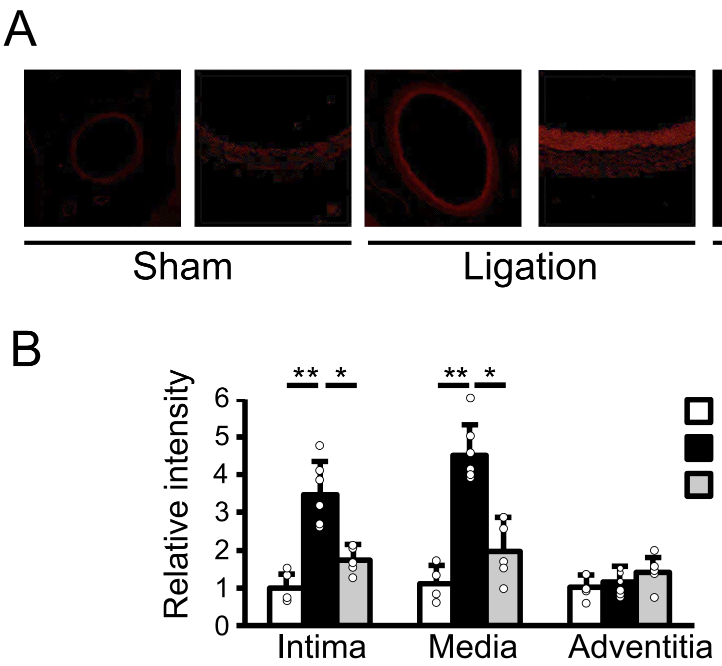

Supplemental Figure 2. Effect of apple polyphenol (AP) treatment on nitrotyrosine levels.
(A) Representative images of immunofluorescence staining of nitrotyrosine.
(B) Relative intensity of immunofluorescence staining. Data are expressed as mean and SD. n = 4 for Sham group, n = 6 for Ligation group, n = 5 for AP group. * $P < 0.05$ ** $P < 0.01$.
